# Quantifying Health Policy Uncertainty in China Using Newspapers: Text Mining Study

**DOI:** 10.2196/46589

**Published:** 2023-11-14

**Authors:** Chen Chen, Junli Zhu

**Affiliations:** 1 School of Public Health Capital Medical University Beijing China; 2 Research Center for Capital Health Management and Policy Capital Medical University Beijing China

**Keywords:** China, health policy, newspaper, uncertainty, severe acute respiratory syndrome, SARS, COVID-19

## Abstract

**Background:**

From the severe acute respiratory syndrome (SARS) outbreak in 2003 to the COVID-19 pandemic in 2019, a series of health measures and policies have been introduced from the central to the local level in China. However, no study has constructed an uncertainty index that can reflect the volatility, risk, and policy characteristics of the health environment.

**Objective:**

We used text mining analysis on mainstream newspapers to quantify the volume of reports about health policy and the total number of news articles and to construct a series of indexes that could reflect the uncertainty of health policy in China.

**Methods:**

Using the Wisenews database, 11 of the most influential newspapers in mainland China were selected to obtain the sample articles. The health policy uncertainty (HPU) index for each month from 2003 to 2022 was constructed by searching articles containing the specified keywords and calculating their frequency. Robustness tests were conducted through correlation analysis. The HPU index was plotted using STATA (version 16.0), and a comparative analysis of the China and US HPU indexes was then performed.

**Results:**

We retrieved 6482 sample articles from 7.49 million news articles in 11 newspapers. The China HPU index was constructed, and the robustness test showed a correlation coefficient greater than 0.74, which indicates good robustness. Key health events can cause index fluctuations. At the beginning of COVID-19 (May 2020), the HPU index climbed to 502.0. In December 2022, China’s HPU index reached its highest value of 613.8 after the release of the “New Ten Rules” pandemic prevention and control policy. There were significant differences in HPU index fluctuations between China and the United States during SARS and COVID-19, as well as during the Affordable Care Act period.

**Conclusions:**

National health policy is a guide for health development, and uncertainty in health policy can affect not only the implementation of policy by managers but also the health-seeking behavior of the people. Here, we conclude that changes in critical health policies, major national or international events, and infectious diseases with widespread impact can create significant uncertainty in China’s health policies. The uncertainty of health policies in China and the United States is quite different due to different political systems and news environments. What is the same is that COVID-19 has brought great policy volatility to both countries. To the best of our knowledge, our work is the first systematic text mining study of HPU in China.

## Introduction

### Background

Health policies are introduced by the government to maintain the stable development of the national health service, but it is difficult for health organizations to know what is included in the policies before they are enacted by the government. Frequent launches of new health policies also lead organizations to worry due to the uncertainty of the expected environment. There are no studies that provide a clear definition of “health policy uncertainty” (HPU). Based on the definition of economic policy uncertainty (EPU) [1], we define HPU as the inability of health care providers to predict exactly whether, when, and how the government will change existing health policies. Uncertainty in the health field persists and has been exacerbated in the wake of the COVID-19 pandemic [2]. Many countries have taken measures, such as keeping social distance and closing down businesses, in response to the pandemic [3,4]. Research has suggested that the COVID-19 pandemic has created almost twice as much economic and health care policy uncertainty as in pre-2020 as countries scramble to respond to an unknown and rapidly evolving threat [5]. The World Health Organization (WHO) states that the development of healthy public policies has been recognized as a cornerstone of new public health [6]. Baker et al [7] suggest that the health care sector is particularly sensitive to HPU, and the risk of policy change is high in sectors such as health care and finance.

In China, 2003 was a turning point in the country’s health care reform. Severe acute respiratory syndrome (SARS) made China realize that a small virus could defeat a nation, and it also made the government realize that the chaos in the health care system would have incalculable consequences. As a result, a large number of policy documents have been issued to promote the rapid development of the public health system. Hospitals are no longer marketized but have returned to being publicized. After the implementation of China’s new health care reform in 2009, systematic and institutional construction began, and the pandemic of COVID-19 since 2019 also led to an explosion of pandemic prevention policies. For more than a decade, a series of reform measures and policies issued from the central to the local level have resulted in substantial changes in the general environment of China’s health care services market.

In recent years, the crisis of health insurance revenue and expenditure caused by the deepening of China’s aging population, the disappearance of demographic advantage due to the decline of the newborn population, and the medium-to-low-speed development of the economy have all made the development of China’s health environment unpredictable. The economic downturn caused by COVID-19 has limited health care investment, and the government will inevitably play the game again between the marketization and publicization of hospitals and carry out a series of reforms to improve the efficiency of the health care sector’s efficiency. National health policy is the guide for health development, and wavering or frequent changes in policy can create uncertainty and make the health environment volatile. Health organizations do not know whether, when, and how the government will change existing policies in the future, which affects the strength and effectiveness of health organizations in implementing policies [8], as well as people’s health-seeking behavior [9]. Bryant [10] indicates that policy changes can affect health promotion and public health. Wiemann and Lumsdaine [2] also suggest that HPU can affect the investment choices of risk-averse households. Therefore, it is necessary to pay attention to the characteristics and changing regulations of health policy.

### Related Works

Baker et al [7] indicated that economic uncertainty consists of real economic uncertainty and EPU. Since the international financial crisis in 2008, EPU has accounted for a rather high share of economic uncertainty. Therefore, they constructed a new EPU index for the first time using the frequency of newspaper reports in the case of the United States [7]. This is an interesting method in which the index is calculated by measuring the frequency of articles containing the keywords “economy,” “uncertainty,” and “policy” in 10 mainstream US newspapers. After that, Azqueta-Gavaldón [11] designed an unsupervised algorithm that automatically recovers the themes of each article, which is less costly and more flexible. Ghirelli et al [12] constructed the Spanish EPU index using the method of Baker et al [7]. Husted et al [13] used the same method to construct the monetary policy uncertainty index. Besides, Baker et al [7] also constructed the US HPU index with the keywords “health care,” “hospital,” and “health insurance,” which provide us with methodological guidance for constructing the HPU index in China. Constructing such an HPU index for China is desirable for the following reasons: first, since the launch of a new round of health care reform in 2009, the Chinese government has embarked on an unprecedented transformation of the health system, and the ensuing shocks of policy uncertainty may have important implications for China’s macroeconomic regulation [14]. In addition, as an emerging economy with better population health compared to other emerging countries [15], the HPU experienced by China may provide a reference for similar countries.

The current constructed index on Chinese policy uncertainty mainly focuses on economics, such as the China EPU index constructed by Baker et al [7] using the “South China Morning Post,” an English-language newspaper published in Hong Kong, China, as a sample. However, the index constructed by only 1 newspaper may be influenced by the editorial policies and preferences of that newspaper as well as the accuracy of translations. Moreover, Davis et al [16] used the “People’s Daily and Guangming Daily” as samples to construct a new EPU index for China through more logical hierarchical keywords. Huang and Luk [14] expanded the sample of newspapers to 10 and referred to the keywords in the study of Davis et al [16] to construct a more accurate EPU index for China. In addition to the EPU index, Yang et al [17] constructed the China Real Estate Policy Uncertainty index, referencing the above method. The above study provides us with an overview of several influential newspapers in China and serves as a reference for the selection of our sample newspapers.

### Objectives

Research focusing on HPU in China is extremely limited. Chen [18] examines health policies that have had a significant impact on the health workforce since the founding of the country by reviewing the literature. Wang et al [19] used public data to assess China’s previous health care reform policies to identify the challenges to achieving a high-quality community health care system. In the postpandemic era, health policies even have an impact on travel and employment [20-22], yet no studies have systematically constructed the HPU index to reflect HPU. For government departments, quantifying HPU can help them understand the degree of social reaction to different policies that have been introduced over time. Higher fluctuations indicate that the policy has created a greater social stir, which can serve as a reference for the number and intensity of policies to be issued in the future. For health care organizations, assessing HPU can help managers understand the organization’s external environment. In addition, managers can make decisions in the current policy environment by reviewing the organization’s implementation of the policy at different times in the past. For the public, the index can reflect the degree of conversation among the residents after the release of the policy, which can reflect the fulfillment of the policy from the side. In terms of research sense, based on the constructed index, scholars can further explore the influence mechanism between HPU and government macrocontrol behaviors such as health fiscal spending. In addition, the impact of HPU on the use of hospital resources and people’s health-seeking behavior can be analyzed at the microlevel. For these reasons, we aim to construct the China HPU index to reflect the volatility and risk of China’s health environment and its policy characteristics.

## Methods

### Properties of the Index

We relied on newspaper articles rather than policy documents to measure policy uncertainty for the following reasons: first, policies usually appear as plain text and are difficult to measure numerically, and second, policy documents could reflect policy changes, whereas the uncertainty caused by the introduction of a policy was the perception of the external environment by policy receivers. This perception could be quantified through the discussion and reaction of the media and the public. The introduction of policies is often accompanied by a high frequency of discussion in the news media, which is often documented. Numerous scholars have used textual statistics to build a policy-related index [14,23,24]. The research of Baker et al [7] indicated a strong correlation between the qualitative index constructed based on news texts and the corresponding quantitative index, which justified the research methodology. Therefore, this study aims to reveal the uncertainty of China’s health policy at different times by counting the number of news articles discussing HPU in mainstream Chinese newspapers.

### Define the Newspaper and Keywords

Considering the professionalism, continuity, authority, circulation range, and data availability of newspapers in mainland China, the Wisenews database listed 27 of the most influential newspapers in China. A total of 11 sample newspapers were selected for this study, including “People’s Daily,” “Guangming Daily,” “Southern Metropolis Daily,” “Yangcheng Evening News,” “New Express Daily,” “Xinmin Evening News,” “Morning News,” “Nanfang Daily,” “Morning Post,” “Beijing Evening News,” and “Beijing Daily.” The sample newspapers were comprehensive, including party newspapers, metropolitan newspapers, national newspapers, and local newspapers. They were also available for 2003 or earlier, and the data were more complete. The second step was to define the keywords. The keywords were grouped into 3 categories, namely “health,” “policy,” and “uncertainty.” Then, we extended the 3 keywords to define the secondary keyword set. Secondary keywords were synonyms or derivatives of the primary keywords. The Chinese keywords and their English translations are presented in Table 1. The sample articles to be screened should cover the following keywords: (1) “health” (or variant); (2) at least 1 of the following terms: “policy” (or variant), “government” (or variant), “country leaders,” “regulation,” or “health care reform”; and (3) at least 1 of the following terms: “uncertainty” (or variant), “volatile,” “unstable” (or variant), “unpredictable,” “plunge,” or “surge.” Baker et al [7], Huang and Luk [14], and Luk et al [24] also used similar methods to define keywords when constructing the EPU index for China. We chose 2003, the year of the SARS outbreak, as the starting year for this search and collected the data for each newspaper on a month-by-month basis from January 2003 to December 2022.

**Table 1:** Chinese keywords in English and Chinese for compiling the health policy uncertainty index.

Criteria and English keywords		Keywords in Chinese
**Health**		
	Health care/medicine	卫生/医药
**Policy**		
	Policy/measures	制度/体制/战略/措施/条例
	Government/authority	政府/国务院/人大/人民代表大会
	Country leaders	主席/总书记/总理
	Regulation	整治/监管/规章
	Health care reform	医改/医疗改革/医保
**Uncertainty**		
	Uncertainty/uncertain	不确定/不明确
	Volatile	波动/不稳
	Unstable/unclear	不明朗/未明
	Unpredictable	难料/难以预计/难以预测/难以估计/难以预料
	Plunge/surge	流失/亏损/骤跌/衰退/激增/滞后

### Target Article Accuracy Test

To ensure the accuracy of the target article selection criteria, a sampling test was used to check whether the machine-selected articles met the keyword set criteria and whether the semantics of the articles conveyed information about the uncertainty of China’s health policies. We randomly selected half of the target articles from each paper and checked the articles for compliance by reading them manually. The auditor was a student at Capital Medical University and was trained before the audit began. The training was conducted in order to clarify the definition of HPU and standardize the criteria for the sample articles. The randomly selected articles were then carefully read under the guidance of weekly team meetings to assess whether the articles were about HPU. To ensure data quality, articles were assessed twice. Articles with inconsistent results between the 2 reviews were then fully discussed and verified to ensure the reliability of the data.

### Construction of the Index

To avoid the impact that the overall volume of articles in different newspapers and different months may have on the index, the raw data needed to be standardized and normalized. We standardized each monthly newspaper-level series to the unit SD from 2003 to 2022 and then averaged across the 11 papers by month. Finally, we normalized the 11-paper series to a mean of 100 from 2003 to 2022. The index was calculated according to the following steps: (1) the number of target reports from 11 newspapers for each month from 2003 to 2022 was collected and denoted as A*_it_*, where *i* represents different newspapers and *t* represents different months; (2) to be precise, let X*_it_* denote the scaled HPU frequency counts for newspaper *i* = 1, 2, ...11 in month *t*, and X*_it_* = A*_it_* / B*_it_*, where B*_it_* is the total number of reports per newspaper for each month; (3) X is normalized to produce a new sequence Y*_it_*, that is, Y*_it_* = X*_it_* / σ*_i_*, where σ*_i_* is the SD of X*_it_*; (4) computing the average of each month’s Y*_it_* for each newspaper to generate the series Z*_t_*; (5) computing M, the mean value of Z*_t_*, and then multiplying all Z*_t_* by (100 / M) to obtain the normalized HPU time-series index.

The HPU index needed to be checked for robustness after it had been constructed. Huang and Luk [14], Yang et al [17], and Luk et al [24] suggested that the constructed indexes do not depend on the selection of sample newspapers by dividing the newspapers into 2 groups and then constructing the EPU indexes separately and testing the correlation between them. The correlation coefficient obtained by Luk et al [24] is the smallest at 0.74, which they consider to be a reliable indication of the robustness of the index. Therefore, similar to Yang et al [17], this study used 0.74 as a criterion to determine whether the index is sufficiently robust. Of the 11 newspapers, 5 have complete data from 2003 to 2022, and 6 have missing data for 2006. Robustness tests were performed as follows. (1) We first calculated the *HPUtotal* index using the 5 newspapers with complete data. (2) We then removed the 2006 data for the 5 newspapers with complete data to match the time intervals with the other 6 and then calculated the *HPUex06* index for 11 newspapers. (3) We then removed the 2006 *HPUtotal* index and performed robustness tests with the *HPUex06* index. If there was no systematic bias in the 2 indexes, the 2006 data from the *HPUtotal* index could be referenced to the *HPUex06* index to initially generate the *HPUfinal* index. (4) Finally, we tested the correlation relationship between the *HPUfinal* index and the *HPUtotal* index; if there was no systematic bias, it means that it is acceptable for the *HPUfinal* index to refer to the *HPUtotal* index 2006 data. The *HPUfinal* index could be used as the final China HPU index.

Then, we performed the robustness test by dividing the newspapers into different groups and testing the correlations among the indexes constructed by each group of newspapers. The first group included 9 newspapers after deleting “Beijing Evening News” and “Beijing Daily” in order to exclude the influence of local media on the robustness of the results. The second and third groups were party newspapers and metropolitan newspapers, respectively, in order to test the effect of the marketability of newspapers on the robustness of the results. Finally, the China HPU index was plotted using STATA software (version 16.0; StataCorp LLC).

### Ethical Considerations

Institutional review board approval was not required for this study since all information was based on open-access published newspaper articles and excluded personal blogs, editorials, and commentaries.

## Results

### Audit of Targeted Articles

We retrieved 6482 targeted articles from 11 newspapers for the period from 2003-2022. “People’s Daily,” “Guangming Daily,” and “Nanfang Daily” had more target articles. The number of target articles per newspaper per year is available in Multimedia Appendix 1. Half of the target articles from each newspaper were randomly selected for quality check, and the passing rate is shown in Multimedia Appendix 2. “Guangming Daily” had the highest passing rate of 91.7% (483/527), followed by “Southern Metropolis Daily” and “Nanfang Daily” with 91.3% (348/381) and 91.3% (650/712), respectively. The audit passing rate for the “Xinmin Evening Daily” was relatively low at 82% (82/100). We considered this passing rate acceptable, suggesting that the vast majority of the retrieved target articles were able to reflect the uncertainty of health policy.

### Robustness

The HPU index constructed in this study from January 2003 to December 2022 is available in Multimedia Appendix 3. The results of the robustness tests are presented in Table 2. The correlation between *HPUex06* and *HPUtotal* was 0.921, and the correlation between *HPUfinal* and *HPUtotal* was 0.923, both of which had no systematic bias, indicating that it was acceptable for the *HPUfinal* index to refer to the *HPUtotal* index for 2006. The *HPUfinal* index could be used as the final China HPU index.

The results of the subgroup robustness tests are shown in Table 3. The correlation between the HPU index and the other 3 groups of indexes was high, at more than 0.8. The correlation between the party and metropolitan newspapers was 0.74, and the robustness was acceptable. The results indicated that the constructed index does not depend on the selection of the newspaper sample. To include more newspapers to ensure the comprehensiveness and reliability of the data, the index constructed by 11 newspapers was still used as the final index.

**Figure 1:**
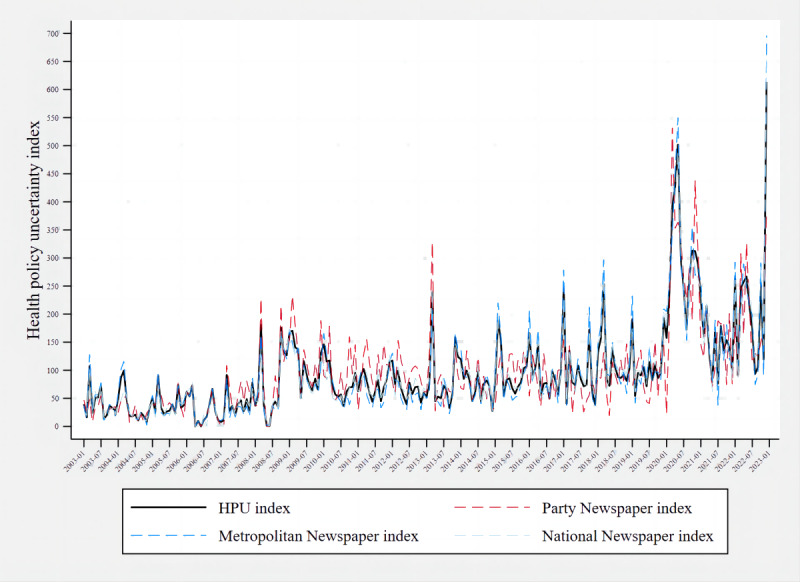
Health policy uncertainty (HPU) index by group.

The indexes of each group are shown in Figure 1. The visual comparison of the line graphs also revealed that the trend changes in each group of indexes were generally consistent.

**Table 2:** Robustness test of the health policy uncertainty (HPU) indexes.

Index		HPUex06	HPUtotal
**HPUex06**			
	*r*	1	0.921
	*P* value	—^a^	<.001
**HPUtotal**			
	*r*	0.921	1
	*P* value	<.001	—^a^
**HPUfinal**			
	*r*	—^a^	0.923
	*P* value	—^a^	<.001

^a^Not applicable.

**Table 3:** Subgroup robustness tests.

Group (newspapers included)		HPU^a^	NNI^b^	PNI^c^	MNI^d^
**HPU (all 11 newspapers)**					
	*r*	1	0.985	0.842	0.986
	*P* value	—^e^	<.001	<.001	<.001
**NNI (** **People’s Daily, Guangming Daily, Southern Metropolis Daily, Yangcheng Evening News, New Express Daily, Xinmin Evening News, Morning News, Nanfang Daily, and Morning Post)**					
	*r*	0.985	1	0.834	0.970
	*P* value	<.001	—	<.001	<.001
**PNI (** **People’s Daily and Guangming Daily)**					
	*r*	0.842	0.834	1	0.740
	*P* value	<.001	<.001	—	<.001
**MNI (** **Southern Metropolis Daily, Yangcheng Evening News, New Express Daily, Xinmin Evening News, Morning News, Nanfang Daily, Morning Post, Beijing Evening News, and Beijing Daily)**					
	*r*	0.986	0.970	0.740	1
	*P* value	<.001	<.001	<.001	—

^a^HPU: health policy uncertainty.

^b^NNI: national newspaper index.

^c^PNI: party newspaper index.

^d^MNI: metropolitan newspaper index.

^e^Not applicable.

### Properties of the Index

**Figure 2:**
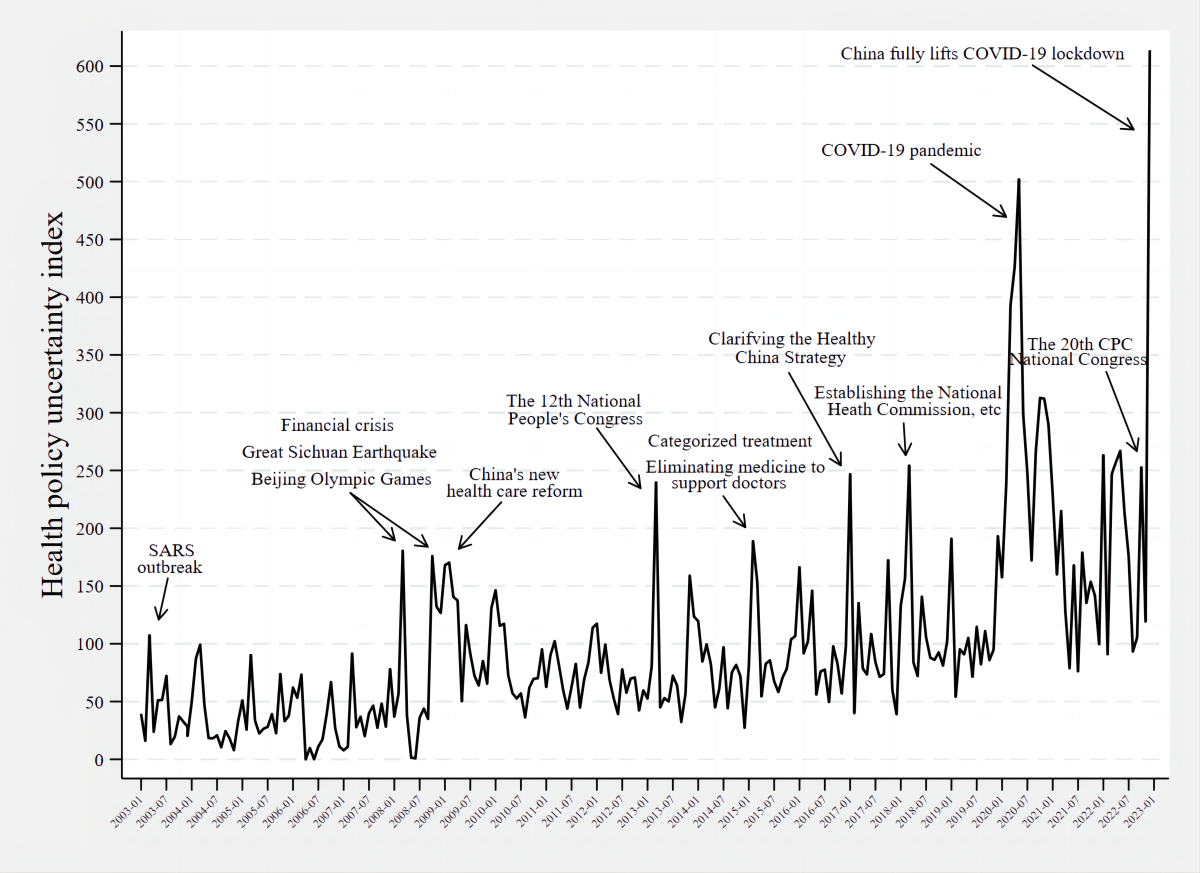
Monthly China health policy uncertainty index from January 2003 to December 2022 with annotated events. CPC: Communist Party of China; SARS: severe acute respiratory syndrome.

Figure 2 shows the HPU index and highlights key health or political events to aid interpretation. The index reflected the following information:

1. Changes in key Chinese health policies, such as the new medical and health system reform in 2009, the categorized treatment and elimination of medicine to support doctors in 2015, and the Health China Strategy proposed in 2016
2. Major national and international events, such as the Sichuan earthquake, the Olympic Games, the economic crisis in 2008, the 12th National People’s Congress in 2013, and the establishment of the National Health Commission and other departments in 2018
3. Infectious diseases with global public health and economic impact, namely, SARS and COVID-19

On December 7, 2022, China issued the “Further Optimization of the Implementation of Measures to Prevent and Control COVID-19,” commonly known as the “New Ten Rules.” This was followed by the nationwide lifting of the pandemic lockdown policy, with the HPU index reaching the highest level in December.

It was notable that the fluctuation of the curve during SARS was not very significant. The 20th National Congress of the Communist Party of China (CPC) in 2022 also did not raise much HPU.

### Comparison With the US HPU Index

**Figure 3:**
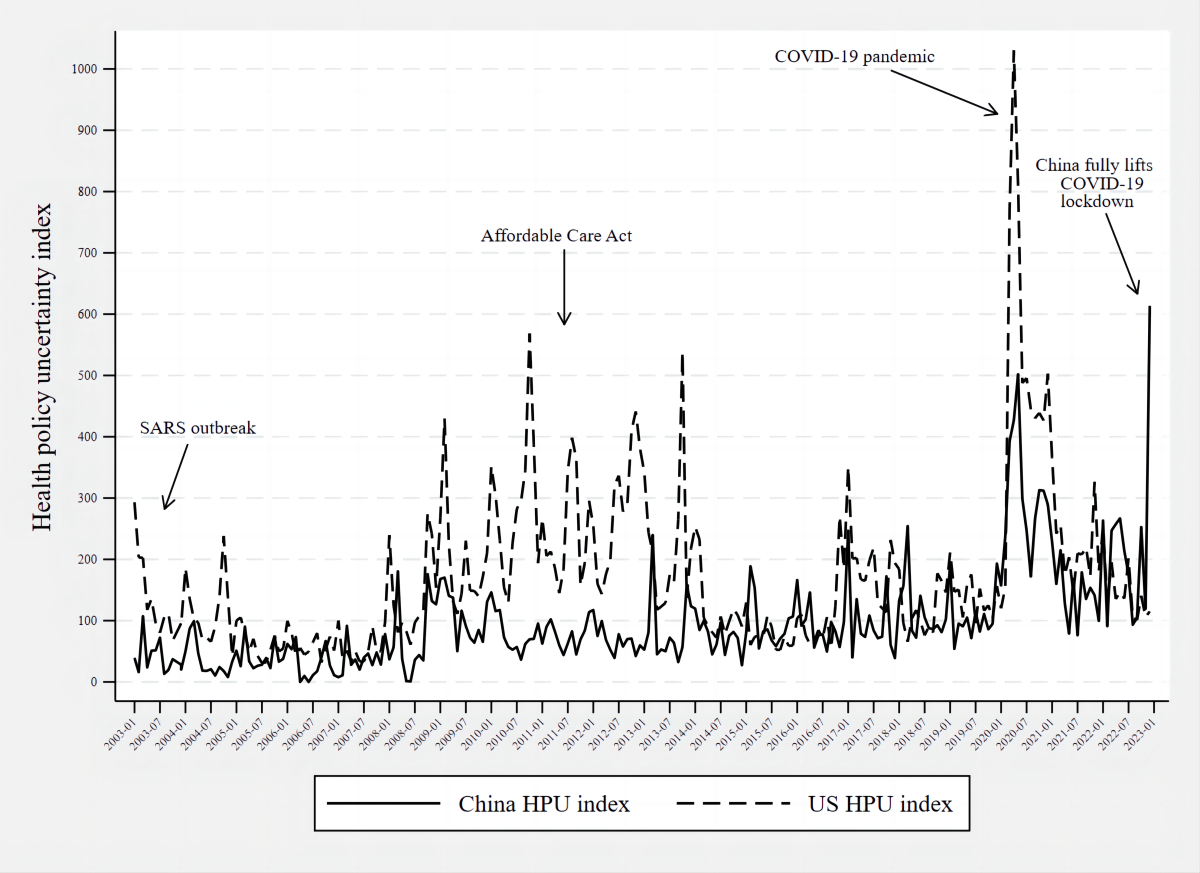
Health policy uncertainty (HPU) index in China and the United States. SARS: severe acute respiratory syndrome.

Comparing our Chinese HPU index with the US HPU index constructed by Baker et al [25,26], the robustness test results indicated a correlation of 0.524 between the 2 groups of indexes. Both indexes peaked during SARS in 2003 and COVID-19 in early 2020, and the US HPU index was higher than China’s around the time of the Affordable Care Act. When China liberalized pandemic prevention and control measures in December 2022, China’s HPU index was higher than that of the United States. Figure 3 presents the HPU index for both the United States and China.

## Discussion

### Overview

In this work, we used text mining to study reports about HPU in the most influential newspapers in China. We retrieved 6482 sample articles from 7.49 million news articles in 11 newspapers and constructed an index of HPU in China by calculating the frequency of the sample newspapers. We found that changes in critical health policies, major national or international events, and infectious diseases with widespread impact can create significant uncertainty in China’s health policies, with the release of the “New Ten Rules” pandemic prevention and control policies leading to the highest value of the HPU index in China. Finally, we compared the HPU indexes of China and the United States. The 2 sets of indexes were very different due to the different political systems and news environments in the 2 countries.

### Characteristics of the China HPU Index

We have found 3 types of events that tend to cause fluctuations in the HPU index, and the first is adjustments in health policy. In 2009, the State Council launched a new round of health care reform, the aim of which was to reduce the burden of health care on residents and achieve full coverage of basic health care. In 2016, the Chinese government also published the “Health China 2030” blueprint. Unlike the health care reform of the past, the “Healthy China Strategy” focuses on a wide range of areas that determine the health of the population, such as lifestyle, air quality, and food security [27]. Such key health policies have been followed by a series of measures and supporting policies [28,29].

The second is a major national or international event. In 2008, in addition to being affected by the global economic crisis, China was struck by the Sichuan earthquake and hosted the 2008 Olympic Games. Mladovsky et al [30] suggested that in response to economic shocks, European policy makers had cut back on essential public health spending, and essential health care had been difficult to maintain due to disruptions in public revenue sources. The Chinese government also announced a series of social policies in the wake of the economic crisis, including spending an additional ¥850 billion (US $123 billion) over 3 years to establish a universal health care system [31]. Besides, the State Council held several meetings after the Sichuan earthquake to decide on disaster relief and postdisaster development measures, and during the Olympic Games, national and local governments also established new policies to guide the public in physical exercise for health [32-34]. In terms of national political events, the 12th National People’s Congress held in March 2013 elected a new national leader, and the change of government provided more opportunities for policy reform, with the uncertainty index rising steeply in that month. In contrast, the election of the 20th Central Committee at the 20th CPC Congress in October 2022 did not bring significant policy uncertainty, probably because Xi Jinping’s successive elections have provided long-term stability and continuity to the Chinese government’s basic policies.

The third is the infectious diseases that have a wide impact. Influenced by SARS in 2003 and COVID-19 in early 2020, the Chinese government has implemented numerous policies to prevent and control the spread of the virus [35,36]. In particular, during COVID-19, the Comprehensive Group of Joint Prevention and Control Mechanisms in China developed a series of policies for the various phases of the pandemic, including public health defense, emergency rescue, and an orderly return to work [37]. On March 11, 2020, the WHO declared that COVID-19 is a pandemic disease, marking the first time that COVID-19 infection has been regarded as a global epidemic, whereas SARS in 2003 did not reach that level [38]. So, the curve fluctuations during SARS were also less prominent compared to COVID-19. Wang [39] analyzed the number of SARS news articles in “People’s Daily” and found that the number of news items was maximized from April 20 to May 31 and then began to decrease and level off in June. This is because the SARS epidemic began in April, and by the end of May, the government had largely brought SARS under control. The uncertainty caused by SARS was short term, and the fluctuations were not significant as the epidemic was well controlled. Furthermore, it is remarkable that the China HPU index reached its highest level in December 2022, even surpassing that of early 2020, when the pandemic first began. The “New Ten Rules” on COVID-19 prevention and control, released on December 7, represent a major change from the policies of the past 3 years, which means that China has entered a new phase of pandemic prevention [40]. It states that negative nucleic acid test certificates and health codes will no longer be checked on people moving across regions, and any form of temporary closure and control will not be allowed. Since then, Beijing, Shanghai, Guangzhou, etc, have issued supporting policies to implement the relevant measures. By January 8, 2023, COVID-19 had changed from category A infectious disease management to category B management.

### Comparison With the US HPU Index

We compared our HPU index with the US HPU index constructed by Baker et al [7] and found that the differences were mainly observed during SARS, COVID-19, and the Affordable Care Act period. On February 11, 2003, the Chinese Ministry of Health declared the SARS outbreak [41]; however, the US HPU index did fluctuate higher than the Chinese HPU index during this period. By July 2003, SARS had spread to over 30 countries and was identified as a global threat to health [42]. Stories of infection in China and warnings to avoid Asia circulated across the United States, and news reports created anxiety and fear [43]. Tian and Stewart [44] suggested that even though the United States did not have a direct outbreak of SARS, its economy, tourism, etc, were affected. We also consider that the volatility of the index is influenced by the source of information in newspapers. Compared to China’s government-dominated news environment [45], government departments, professionals, and the general public are all considered sources of information for US newspapers [41], which may have made the US HPU index more volatile over the same period. Additionally, during this period, the US Congress adopted the Medicare Modernization Act, which has been described as the most important health care legislation of the millennium [46], leading to fluctuations in the US HPU index [2].

There are also significant differences in the HPU index between the 2 countries following the enactment of the Affordable Care Act. Interestingly, China’s new health care reform was also launched during this period [47], and the difference may be due to the different political party systems of the 2 countries [48]. China has a 1-party rule and a multiparty cooperation system. The CPC, as the ruling party, is the direct subject of the most important public policies [49]. The change in China’s government is not a Western-style replacement of political parties but a change of the old and the new at the end of the term of office. The basic line of the CPC does not change with a change of government or with a change of leaders. So, the basic policies of the Chinese government have stability and continuity. Thus, even when China elected a new national leader in 2013, the HPU index only increased significantly in the month of the election campaign, and Xi’s reelection in October 2022 did not bring much volatility. The United States, however, has a 2-party system. The external competitive pressure leads both parties to take an active interest in public policy issues to gain public political support [50]. This system also results in a lack of continuity and stability in policy-making in the United States [48]. Even though both countries have implemented important health care policies during this period, policy uncertainty will be higher in the United States than in China. Wiemann and Lumsdaine [2] also suggested that, more than a decade after the enactment of the Affordable Care Act, health care policy remains a central topic of political debate in the United States.

The third peak occurs during COVID-19, and it suggests that HPU in the United States during COVID-19 appears to be higher than in China. Although the first case of COVID-19 detected in China was reported by the WHO on December 31, 2019, China controlled COVID-19 earlier due to the immediate implementation of a strict prevention and control isolation policy [51]. In contrast, the pandemic was more complex in the United States. The first difference was precautionary actions; while China recommended using masks in public places at the beginning of the pandemic, the United States updated its guidelines to require masks and social distancing sometime after the pandemic had broken out [52,53]. However, this measure was poorly implemented due to political beliefs [54]. The second difference was the screening criteria; although information about contact with suspected and confirmed cases was essential for case screening, this was not included in the United States. The United States also does not include travel history as a criterion [52]. In China, all people suspected of having a COVID-19 infection were instructed to seek testing at a testing center and were admitted to a “Fangcang shelter hospital.” There were also problems with the quality and availability of COVID-19 testing kits in the early stages of the pandemic in the United States, which further added to the uncertainty of the environment [52].

### Limitations and Future Directions

This study is not without its limitations. First, influenced by the social system, newspapers in China are owned by the government and managed mainly through administrative means. US newspapers, on the other hand, have more diverse sources of information, so the HPU indexes of the 2 countries are simply for comparison. Second, the layout and the news frame of reports are also important indicators reflecting the media’s attitude toward public health issues, yet these indicators are not available because the data started too early. Third, it can be seen that the China HPU index reached its highest point in December 2022, and it is not known whether it will continue to increase or decrease thereafter. However, more measures and news reports are expected to follow, and the study will continue to track the latest data. Finally, some of the target articles do not meet the sample requirements, and we will explore more advanced methods to improve the accuracy of identifying HPU. The direct link between events and uncertainty can be verified through causal inference in subsequent studies.

### Practical Significance

This study is the initial attempt to construct an index of HPU in China. The results show that the HPU index constructed in this study is reasonable and can reflect the uncertainty of health policy in China to a certain extent. Our index is updated to December 2022, when China issued its “New Ten Rules” pandemic prevention policy. This policy has important implications for China’s pandemic control and even economic development, etc (which is also reflected in the volatility of the index). However, there are currently no domestic or international studies discussing this policy, and we will continue to track the changes in the HPU index. In the postpandemic era, recognizing the changing environment of health policy is beneficial for managers to make an accurate assessment of market conditions and make effective and correct decisions. In addition, the consideration of policy uncertainty is also beneficial to the government in making rational decisions and judgments on whether to introduce policies and the effects of policies after implementation, and it is a guide to the formulation and introduction of policies in the future.

### Conclusion

We selected data from 11 of the most influential newspapers in mainland China to construct the China HPU index for the period from January 2003 to December 2022. The robustness test results indicate that the HPU index constructed in this study is reasonable and can reflect the uncertainty of China’s health policy to a certain extent, such as the timing, frequency, and strength of policy introduction. In addition, comparing our constructed China HPU index with the US HPU index, we find that there are significant differences between the 2 countries during SARS and COVID-19 and during the Affordable Care Act period.

## Appendix

Multimedia Appendix 1 The number of target articles per newspaper per year.

Multimedia Appendix 2 Results of the target article accuracy audit.

Multimedia Appendix 3 China health policy uncertainty index.

## Abbreviations

CPC: Communist Party of China
EPU: economic policy uncertainty
HPU: health policy uncertainty
SARS: severe acute respiratory syndrome
WHO: World Health Organization

## Data Availability

The data sets generated during this study have been uploaded as multimedia appendices.
