# Supplementary material for: Quantifying Health Policy Uncertainty in China Using Newspapers: Text Mining Study
Source: J Med Internet Res. 2023 Nov 14;25:e46589. doi: 10.2196/46589 (PMC10685290; doi:10.2196/46589)
Supplement: Multimedia Appendix 1 [file jmir_v25i1e46589_app1.docx]

**Multimedia Appendix 1**

**Table S1.** The number of target articles per newspaper per year.

|  | People's Daily | Guangming Daily | Southern Metropolis Daily | Yangcheng Evening News | New Express Daily | Xinmin Evening News | Morning News | Nanfang Daily | Morning Post | Beijing Evening News | Beijing Daily |
| --- | --- | --- | --- | --- | --- | --- | --- | --- | --- | --- | --- |
| 2003 | 36 | 18 | 4 | 15 | 6 | 9 | 1 | 2 | 2 | 4 | 17 |
| 2004 | 43 | 7 | 15 | 8 | 11 | 6 | 4 | 16 | 2 | 4 | 26 |
| 2005 | 38 | 26 | 33 | 8 | 2 | 5 | 1 | 15 | 7 | 4 | 22 |
| 2006 | - | - | - | 7 | 8 | - | 0 | - | - | 2 | 14 |
| 2007 | 36 | 42 | 32 | 4 | 8 | 5 | 3 | 49 | 1 | 5 | 21 |
| 2008 | 62 | 58 | 61 | 13 | 5 | 11 | 6 | 106 | 8 | 9 | 25 |
| 2009 | 111 | 79 | 105 | 15 | 15 | 17 | 16 | 141 | 7 | 7 | 69 |
| 2010 | 102 | 50 | 93 | 6 | 7 | 5 | 3 | 111 | 8 | 18 | 37 |
| 2011 | 115 | 51 | 80 | 13 | 7 | 9 | 10 | 116 | 6 | 6 | 25 |
| 2012 | 93 | 52 | 13 | 11 | 5 | 11 | 11 | 53 | 8 | 4 | 20 |
| 2013 | 89 | 41 | 27 | 26 | 17 | 8 | 17 | 54 | 13 | 6 | 20 |
| 2014 | 70 | 48 | 25 | 20 | 14 | 10 | 6 | 57 | 3 | 6 | 29 |
| 2015 | 99 | 66 | 28 | 16 | 22 | 10 | 5 | 50 | 7 | 13 | 15 |
| 2016 | 87 | 44 | 20 | 13 | 13 | 10 | 9 | 65 | 2 | 12 | 21 |
| 2017 | 66 | 39 | 28 | 17 | 12 | 8 | 3 | 73 | 2 | 10 | 25 |
| 2018 | 68 | 52 | 33 | 20 | 11 | 10 | 2 | 67 | 5 | 10 | 18 |
| 2019 | 59 | 49 | 46 | 11 | 18 | 4 | 0 | 78 | 4 | 10 | 20 |
| 2020 | 155 | 182 | 63 | 26 | 35 | 33 | 9 | 173 | 1 | 23 | 74 |
| 2021 | 98 | 56 | 14 | 24 | 9 | 16 | 4 | 83 | 1 | 10 | 42 |
| 2022 | 126 | 94 | 42 | 20 | 5 | 12 | 11 | 115 | 4 | 10 | 42 |
