# Supplementary figures and images for: Quantifying Health Policy Uncertainty in China Using Newspapers: Text Mining Study
Source: J Med Internet Res. 2023 Nov 14;25:e46589. doi: 10.2196/46589 (PMC10685290; doi:10.2196/46589)

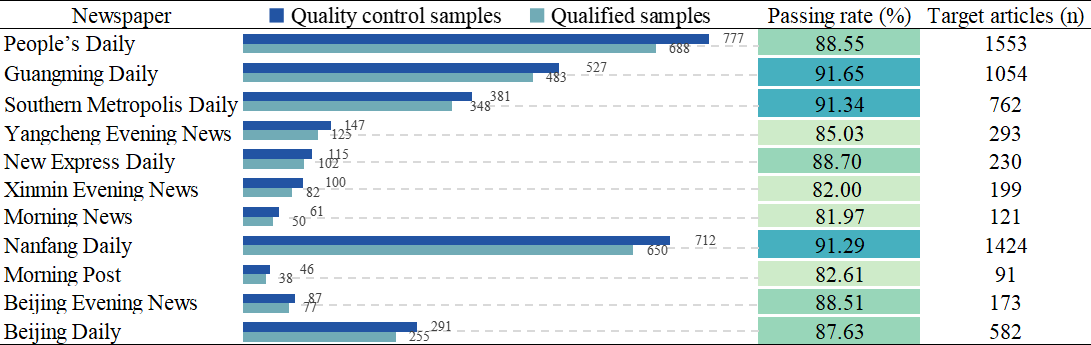

Supplement: Multimedia Appendix 2 [file jmir_v25i1e46589_app2.png]
